# Supplementary material for: Hawaiian black coral (Antipatharia) complete mitochondrial genomes have limited phylogenetic signal for taxonomic resolution of species
Source: PeerJ. 2025 May 30;13:e18731. doi: 10.7717/peerj.18731 (PMC12129002; doi:10.7717/peerj.18731)
Supplement: Supplemental Information 1 [file peerj-13-18731-s001.docx]

**Hawaiian black coral (Antipatharia) complete mitochondrial genomes have limited phylogenetic signal for taxonomic resolution of species**

Supplementary Tables

Table S1. GenBank verified sequence accession numbers for ʻēkaha kū moana (Hawaiian black coral) samples used in this study. Antipathes griggi #196 represents type material stored at the Bernice Pauahi Bishop Museum (BPBM).

| **Species name** | **Voucher ID** | **GenBank Verified Sequences Accession Numbers** |
| --- | --- | --- |
| *Antipathes grandis* | 168 | PP498838 (complete mitogenome) |
| *Antipathes grandis* | 187 | PP506421 (complete mitogenome) |
| *Antipathes griggi* | 176 | PV357613 (ATP6), PV357614 (ATP8), PV339451 (CO1), PV357615 (COX2), PV357616 (COX3), PV357617 (CYTB), PV357618 (HEG), PV357619 (ND1), PV357620 (ND2), PV357621 (ND3), PV357622 (ND4), PV357623 (ND4L), PV357624 (ND5), PV357625 (ND6) |
| *Antipathes griggi* | 196 | PV357626 (ATP6), PV357627 (ATP8), PV344502 (CO1), PV357628 (COX2), PV357629 (COX3), PV357630 (CYTB), PV357631 (HEG), PV357632 (ND1), PV357633 (ND2), PV357634 (ND3), PV357635 (ND4), PV357636 (ND4L), PV357637 (ND5), PV357638 (ND6) |
| *Aphanipathes verticillata* | 130 | PV390671 (ATP6), PV390670 (ATP8), PV360965 (CO1), PV390667 (COX2), PV390665 (COX3), PV390674 (CYTB), PV390673 (ND3), PV390668 (ND4), PV390666 (ND4L), PV390672 (ND5), PV390669 (ND6) |
| *Cirrhipathes* cf. *anguina LS-2022* | 179 | ON653414 (complete mitogenome) |
| *Cirrhipathes* cf. *anguina* | 361 | PV390682 (ATP6), PV390681 (ATP8), PV364093 (CO1), PV390678 (COX2), PV390675 (COX3), PV390687 (CYTB), PV390676 (HEG), PV390684 (ND1), PV390686 (ND2), PV390685 (ND3), PV390679 (ND4), PV390677 (ND4L), PV390683 (ND5), PV390680 (ND6) |
| *Myriopathes ulex* | 144 | OP104910 (complete mitogenome) |
| *Myriopathes* cf. *ulex* | 269 | PV390688 (ATP6), PV390689 (ATP8), PV361221 (CO1), PV390690 (COX2), PV390691 (COX3), PV390692 (ND3), PV390693 (ND4L), PV390694 (ND5) |
| *Stichopathes* cf. *maldivensis* | 180 | PV357600 (ATP6), PV357601 (ATP8), PV194956 (CO1), PV357602 (COX2), PV357603 (COX3), PV357604 (CYTB), PV357605 (HEG), PV357607 (ND2), PV357608 (ND3), PV357609 (ND4), PV357610 (ND4L), PV357611 (ND5), PV357612 (ND6) |

Table S2. Mitogenome annotations of *Antipathes* *grandis* (#168)

| **Name** | **Type** | **Start** | **End** | **Length** | **Transferred From** | **Transferred Similarity** |
| --- | --- | --- | --- | --- | --- | --- |
| **COX1** | gene | 1  2373 | 894  3701 | 1593 | *Stichopathes* sp. SCBUCN-8850 | 99.74% |
| **intron** | intron | 895 | 2372 | 1478 | *Stichopathes* sp. SCBUCN-8850 | 99.66% |
| **HEG** | gene | 1205 | 2266 | 1062 | *Stichopathes* *luetkeni* | 99.91% |
| **ND4L** | gene | 3220 | 3519 | 300 | *Stichopathes* sp. SCBUCN-8850 | 100.00% |
| **COX2** | gene | 3627 | 4376 | 750 | *Stichopathes* sp. SCBUCN-8850 | 99.87% |
| **ND4** | gene | 4433 | 5935 | 1503 | *Stichopathes* sp. SCBUCN-8850 | 100.00% |
| **ND6** | gene | 6141 | 6731 | 591 | *Stichopathes* sp. SCBUCN-8850 | 99.83% |
| **ATP8** | gene | 6806 | 7018 | 213 | *Stichopathes* sp. SCBUCN-8850 | 99.53% |
| **ATP6** | gene | 7192 | 7890 | 699 | *Stichopathes* sp. SCBUCN-8850 | 99.86% |
| **ND5** | gene | 8029  10811 | 8748  11941 | 1851 | *Stichopathes* sp. SCBUCN-8850 | 100.00% |
| **intron** | intron | 8749 | 10810 | 2062 | *Stichopathes* sp. SCBUCN-8850 | 99.66% |
| **ND1** | gene | 9099 | 10148 | 1050 | n/a | n/a |
| **ND3** | gene | 10286 | 10642 | 357 | n/a | n/a |
| **tRNA** | tRNA | 11986 | 12055 | 70 | *Stichopathes* sp. SCBUCN-8850 | 100% |
| **ND2** | gene | 12175 | 13692 | 1518 | *Stichopathes* sp. SCBUCN-8850 | 99.80% |
| **rRNA** | rRNA | 13922 | 15070 | 1149 | *Stichopathes* sp. SCBUCN-8850 | 99.91% |
| **CYTB** | gene | 15176 | 16318 | 1143 | *Stichopathes* sp. SCBUCN-8850 | 99.83% |
| **tRNA** | tRNA | 16395 | 16465 | 71 | *Stichopathes* sp. SCBUCN-8850 | 100.00% |
| **rRNA** | rRNA | 16570 | 19232 | 2663 | *Stichopathes* sp. SCBUCN-8850 | 99.81% |
| **COX3** | gene | 19400 | 20188 | 789 | *Stichopathes* sp. SCBUCN-8850 | 99.75% |

Table S3. Mitogenome annotations of *Antipathes* *grandis* (#187)

| **Name** | **Type** | **Start** | **End** | **Length** | **Transferred From** | **Transferred Similarity** |
| --- | --- | --- | --- | --- | --- | --- |
| **COX1** | gene | 1  2373 | 894  3071 | 1593 | *Stichopathes* sp. SCBUCN-8850 | 99.74% |
| **intron** | intron | 895 | 2372 | 1478 | *Stichopathes* sp. SCBUCN-8850 | 99.66% |
| **HEG** | gene | 1205 | 2266 | 1062 | *Stichopathes luetkeni* | 99.91% |
| **ND4L** | gene | 3220 | 3519 | 300 | *Stichopathes luetkeni* | 100.00% |
| **COX2** | gene | 3627 | 4376 | 750 | *Stichopathes luetkeni* | 99.87% |
| **ND4** | gene | 4433 | 5935 | 1503 | *Stichopathes* sp. SCBUCN-8850 | 100.00% |
| **ND6** | gene | 6141 | 6731 | 591 | *Stichopathes* sp. SCBUCN-8850 | 99.83% |
| **ATP8** | gene | 6806 | 7018 | 213 | *Stichopathes luetkeni* | 99.53% |
| **ATP6** | gene | 7192 | 7890 | 699 | *Stichopathes* sp. SCBUCN-8850 | 99.86% |
| **ND5** | gene | 8029  10811 | 8748  11941 | 1851 | *Stichopathes luetkeni* | 99.72% |
| **intron** | intron | 8749 | 10810 | 2062 | *Stichopathes* sp. SCBUCN-8850 | 99.66% |
| **ND1** | gene | 9099 | 10148 | 1050 | *Stichopathes luetkeni* | 100.00% |
| **ND3** | gene | 10286 | 10642 | 357 | *Stichopathes luetkeni* | 100.00% |
| **tRNA** | tRNA | 11986 | 12055 | 70 | *Stichopathes* sp. SCBUCN-8850 | 100.00% |
| **ND2** | gene | 12175 | 13692 | 1518 | *Stichopathes* sp. SCBUCN-8850 | 99.80% |
| **rRNA** | rRNA | 13922 | 15070 | 1149 | *Stichopathes luetkeni* | 99.91% |
| **CYTB** | gene | 15176 | 16318 | 1143 | *Stichopathes luetkeni* | 99.83% |
| **tRNA** | tRNA | 16395 | 16465 | 71 | *Stichopathes luetkeni* | 100.00% |
| **rRNA** | rRNA | 16570 | 19232 | 2663 | *Stichopathes* sp. SCBUCN-8850 | 99.81% |
| **COX3** | gene | 19400 | 20188 | 789 | *Stichopathes luetkeni* | 99.75% |

Table S4. Mitogenome annotations of *Antipathes* *griggi* (#176)

| **Name** | **Type** | **Start** | **End** | **Length** | **Transferred From** | **Transferred Similarity** |
| --- | --- | --- | --- | --- | --- | --- |
| **COX1** | gene | 1  2370 | 894  3068 | 1593 | *Stichopathes* sp. SCBUCN-8850 | 99.90% |
| **intron** | intron | 895 | 2369 | 1475 | *Stichopathes* sp. SCBUCN-8850 | 99.80% |
| **HEG** | gene | 1202 | 2263 | 1062 | *Stichopathes* sp. SCBUCN-8850 | 100.00% |
| **ND4L** | gene | 3217 | 3516 | 300 | *Stichopathes* sp. SCBUCN-8850 | 100.00% |
| **COX2** | gene | 3624 | 4373 | 750 | *Stichopathes* sp. SCBUCN-8850 | 100.00% |
| **ND4** | gene | 4430 | 5932 | 1503 | *Stichopathes* sp. SCBUCN-8850 | 100.00% |
| **ND6** | gene | 6138 | 6728 | 591 | *Stichopathes* sp. SCBUCN-8850 | 100.00% |
| **ATP8** | gene | 6803 | 7015 | 213 | *Stichopathes* sp. SCBUCN-8850 | 100.00% |
| **ATP6** | gene | 7189 | 7887 | 699 | *Stichopathes* sp. SCBUCN-8850 | 100.00% |
| **ND5** | gene | 8026  10808 | 8745  11938 | 1851 | n/a | n/a |
| **intron** | intron | 8746 | 10807 | 2062 | *Stichopathes* sp. SCBUCN-8850 | 99.95% |
| **ND1** | gene | 9096 | 10145 | 1050 | *Stichopathes* sp. SCBUCN-8850 | 100.00% |
| **ND3** | gene | 10283 | 10639 | 357 | *Stichopathes* sp. SCBUCN-8850 | 100.00% |
| **tRNA** | tRNA | 11983 | 12052 | 70 | *Stichopathes* sp. SCBUCN-8850 | 100.00% |
| **ND2** | gene | 12172 | 13689 | 1518 | *Stichopathes* sp. SCBUCN-8850 | 100.00% |
| **rRNA** | rRNA | 13919 | 15067 | 1149 | *Stichopathes* sp. SCBUCN-8850 | 100.00% |
| **CYTB** | gene | 15173 | 16315 | 1143 | *Stichopathes* sp. SCBUCN-8850 | 100.00% |
| **tRNA** | tRNA | 16392 | 16462 | 71 | *Stichopathes* sp. SCBUCN-8850 | 100.00% |
| **rRNA** | rRNA | 16567 | 19229 | 2663 | *Stichopathes* sp. SCBUCN-8850 | 100.00% |
| **COX3** | gene | 19396 | 20184 | 789 | *Stichopathes* sp. SCBUCN-8850 | 100.00% |

Table S5. Mitogenome annotations of *Antipathes* *griggi* (#196)

| **Name** | **Type** | **Start** | **End** | **Length** | **Transferred From** | **Transferred Similarity** |
| --- | --- | --- | --- | --- | --- | --- |
| **COX1** | gene | 1  2373 | 928  3071 | 1627 | *Stichopathes* sp. SCBUCN-8850 | 100.00% |
| **COX3** | gene | 19398 | 20186 | 789 | *Stichopathes* sp. SCBUCN-8850 | 100.00% |
| **rRNA** | rRNA | 16569 | 19231 | 2663 | *Stichopathes* sp. SCBUCN-8850 | 100.00% |
| **tRNA** | tRNA | 16394 | 16464 | 71 | *Stichopathes* sp. SCBUCN-8850 | 100.00% |
| **CYTB** | gene | 15175 | 16317 | 1143 | *Stichopathes* sp. SCBUCN-8850 | 100.00% |
| **rRNA** | rRNA | 13921 | 15069 | 1149 | *Stichopathes* sp. SCBUCN-8850 | 100.00% |
| **ND2** | gene | 12174 | 13691 | 1518 | *Stichopathes* sp. SCBUCN-8850 | 100.00% |
| **tRNA** | tRNA | 11985 | 12054 | 70 | *Stichopathes* sp. SCBUCN-8850 | 100.00% |
| **ND3** | gene | 10286 | 10642 | 357 | *Stichopathes* sp. SCBUCN-8850 | 100.00% |
| **ND1** | gene | 9099 | 10148 | 1050 | *Stichopathes* sp. SCBUCN-8850 | 100.00% |
| **intron** | intron | 8749 | 10810 | 2062 | *Stichopathes* sp. SCBUCN-8850 | 99.90% |
| **ND5** | gene | 8029  10811 | 8748  11941 | 1851 | n/a | n/a |
| **ATP6** | gene | 7192 | 7890 | 699 | *Stichopathes* sp. SCBUCN-8850 | 100.00% |
| **ATP8** | gene | 6806 | 7018 | 213 | *Stichopathes* sp. SCBUCN-8850 | 100.00% |
| **ND6** | gene | 6141 | 6731 | 591 | *Stichopathes* sp. SCBUCN-8850 | 100.00% |
| **ND4** | gene | 4433 | 5935 | 1503 | *Stichopathes* sp. SCBUCN-8850 | 100.00% |
| **COX2** | gene | 3627 | 4376 | 750 | *Stichopathes* sp. SCBUCN-8850 | 100.00% |
| **ND4L** | gene | 3220 | 3519 | 300 | *Stichopathes* sp. SCBUCN-8850 | 100.00% |
| **HEG** | gene | 1205 | 2266 | 1062 | *Stichopathes* sp. SCBUCN-8850 | 100.00% |
| **intron** | intron | 929 | 2372 | 1444 | *Stichopathes* sp. SCBUCN-8850 | 100.00% |

Table S6. Mitogenome annotations of *Aphanipathes* *verticillata* (#130)

| **Name** | **Type** | **Start** | **End** | **Length** | **Transferred From** | **Transferred Similarity** |
| --- | --- | --- | --- | --- | --- | --- |
| **COX1** | gene | 1  2371 | 894  3069 | 1593 | n/a | n/a |
| **intron** | intron | 895 | 2370 | 1476 | n/a | n/a |
| **ND4L** | gene | 3219 | 3506 | 288 | *Stichopathes* sp. SCBUCN-8850 | 95.67% |
| **COX2** | gene | 3626 | 4375 | 750 | *Stichopathes* sp. SCBUCN-8849 | 96.13% |
| **ND4** | gene | 4432 | 5934 | 1503 | *Stichopathes* sp. SCBUCN-8850 | 95.34% |
| **ND6** | gene | 6138 | 6728 | 591 | *Stichopathes* sp. SCBUCN-8849 | 95.09% |
| **ATP8** | gene | 6805 | 7017 | 213 | *Stichopathes* sp. SCBUCN-8849 & *Stichopathes* sp. SCBUCN-8850 | 96.71% |
| **ATP6** | gene | 7184 | 7882 | 699 | *Stichopathes* sp. SCBUCN-8850 | 93.06% |
| **ND5** | gene | 8016  10777 | 8735  11907 | 1851 | n/a | n/a |
| **intron** | intron | 8736 | 10776 | 2041 | n/a | n/a |
| **ND1** | gene | 9070 | 10096 | 1027 | *Stichopathes* sp. SCBUCN-8850 | 98.00% |
| **ND3** | gene | 10252 | 10608 | 357 | *Stichopathes* sp. SCBUCN-8850 | 96.64% |
| **tRNA** | tRNA | 11947 | 12016 | 70 | *Stichopathes* sp. SCBUCN-8850 | 100.00% |
| **ND2** | gene | 12499 | 13644 | 1146 | *Chrysopathes* *formosa* | 90.84% |
| **rRNA** | rRNA | 13874 | 15024 | 1151 | Stichopathes sp. SCBUCN-8849 | 96.79% |
| **CYTB** | gene | 15132 | 16274 | 1143 | *Stichopathes* sp. SCBUCN-8850 | 94.93% |
| **tRNA** | tRNA | 16352 | 16422 | 71 | *Trissopathes* cf. *tetracrada* NB-202 | 97.18% |
| **rRNA** | rRNA | 16539 | 19180 | 2642 | *Stichopathes* sp. SCBUCN-8850 | 92.59% |
| **COX3** | gene | 19348 | 20136 | 789 | *Stichopathes* sp. SCBUCN-8850 | 94.80% |

Table S7. Mitogenome annotations of *Cirrhipathes* cf. *anguina* (#361)

| **Name** | **Type** | **Start** | **End** | **Length** | **Transferred From** | **Transferred Similarity** |
| --- | --- | --- | --- | --- | --- | --- |
| **intron** | intron | 94 | 1571 | 1478 | *Stichopathes* sp. SCBUCN-8850 | 100.00% |
| **HEG** | gene | 404 | 1465 | 1062 | *Stichopathes* sp. SCBUCN-8850 | 100.00% |
| **COX1** | gene | 19662  1572 | 20462  2270 | 1500 | *Stichopathes* sp. SCBUCN-8850 | 99.90% |
| **ND4L** | gene | 2419 | 2718 | 300 | *Stichopathes* sp. SCBUCN-8850 | 100.00% |
| **COX2** | gene | 2826 | 3575 | 750 | *Stichopathes* sp. SCBUCN-8850 | 100.00% |
| **ND4** | gene | 3632 | 5134 | 1503 | *Stichopathes* sp. SCBUCN-8850 | 100.00% |
| **ND6** | gene | 5340 | 5930 | 591 | *Stichopathes* sp. SCBUCN-8850 | 100.00% |
| **ATP8** | gene | 6005 | 6217 | 213 | *Stichopathes* sp. SCBUCN-8850 | 100.00% |
| **ATP6** | gene | 6391 | 7089 | 699 | *Stichopathes* sp. SCBUCN-8850 | 100.00% |
| **ND5** | gene | 7228  10010 | 7947  11140 | 1851 | n/a | n/a |
| **intron** | intron | 7948 | 10009 | 2062 | *Stichopathes* sp. SCBUCN-8850 | 99.90% |
| **ND1** | gene | 8298 | 9347 | 1050 | *Stichopathes* sp. SCBUCN-8850 | 100.00% |
| **ND3** | gene | 9485 | 9841 | 357 | *Stichopathes* sp. SCBUCN-8850 | 100.00% |
| **tRNA** | tRNA | 11184 | 11253 | 70 | *Stichopathes* sp. SCBUCN-8850 | 100.00% |
| **ND2** | gene | 11373 | 12890 | 1518 | *Stichopathes* sp. SCBUCN-8850 | 100.00% |
| **rRNA** | rRNA | 13120 | 14268 | 1149 | *Stichopathes* sp. SCBUCN-8850 | 100.00% |
| **CYTB** | gene | 14374 | 15516 | 1143 | *Stichopathes* sp. SCBUCN-8850 | 100.00% |
| **tRNA** | tRNA | 15593 | 15663 | 71 | *Stichopathes* sp. SCBUCN-8850 | 100.00% |
| **rRNA** | rRNA | 15768 | 18430 | 2663 | *Stichopathes* sp. SCBUCN-8850 | 100.00% |
| **COX3** | gene | 18597 | 19385 | 789 | *Stichopathes* sp. SCBUCN-8850 | 100.00% |

Table S8. Mitogenome annotations of *Stichopathes* sp. (#180)

| **Name** | **Type** | **Start** | **End** | **Length** | **Transferred From** | **Transferred Similarity** |
| --- | --- | --- | --- | --- | --- | --- |
| **COX1** | gene | 1  2370 | 894  3068 | 1593 | *Stichopathes* sp. SCBUCN-8850 | 100.00% |
| **intron** | intron | 895 | 2369 | 1475 | *Stichopathes* sp. SCBUCN-8850 | 99.80% |
| **HEG** | gene | 1202 | 2263 | 1062 | *Stichopathes* sp. SCBUCN-8850 | 100.00% |
| **ND4L** | gene | 3217 | 3516 | 300 | *Stichopathes* sp. SCBUCN-8850 | 100.00% |
| **COX2** | gene | 3624 | 4373 | 750 | *Stichopathes* sp. SCBUCN-8850 | 100.00% |
| **ND4** | gene | 4430 | 5932 | 1503 | *Stichopathes* sp. SCBUCN-8850 | 100.00% |
| **ND6** | gene | 6138 | 6728 | 591 | *Stichopathes* sp. SCBUCN-8850 | 100.00% |
| **ATP8** | gene | 6803 | 7015 | 213 | *Stichopathes* sp. SCBUCN-8850 | 100.00% |
| **ATP6** | gene | 7189 | 7887 | 699 | *Stichopathes* sp. SCBUCN-8850 | 100.00% |
| **ND5** | gene | 8026  10809 | 8745  11939 | 1851 | n/a | n/a |
| **intron** | intron | 8746 | 10808 | 2063 | *Stichopathes* sp. SCBUCN-8850 | 99.90% |
| **ND1** | gene | 9096 | 10146 | 1051 | *Stichopathes* sp. SCBUCN-8850 | 99.90% |
| **ND3** | gene | 10284 | 10640 | 357 | *Stichopathes* sp. SCBUCN-8850 | 100.00% |
| **tRNA** | tRNA | 11983 | 12052 | 70 | *Stichopathes* sp. SCBUCN-8850 | 100.00% |
| **ND2** | gene | 12172 | 13689 | 1518 | *Stichopathes* sp. SCBUCN-8850 | 100.00% |
| **rRNA** | rRNA | 13919 | 15067 | 1149 | *Stichopathes* sp. SCBUCN-8850 | 100.00% |
| **CYTB** | gene | 15173 | 16315 | 1143 | *Stichopathes* sp. SCBUCN-8850 | 100.00% |
| **tRNA** | tRNA | 16392 | 16462 | 71 | *Stichopathes* sp. SCBUCN-8850 | 100.00% |
| **rRNA** | rRNA | 16567 | 19229 | 2663 | *Stichopathes* sp. SCBUCN-8850 | 100.00% |
| **COX3** | gene | 19396 | 20184 | 789 | *Stichopathes* sp. SCBUCN-8850 | 100.00% |
