# Supplementary material for: Hawaiian black coral (Antipatharia) complete mitochondrial genomes have limited phylogenetic signal for taxonomic resolution of species
Source: PeerJ. 2025 May 30;13:e18731. doi: 10.7717/peerj.18731 (PMC12129002; doi:10.7717/peerj.18731)
Supplement: Supplemental Information 3 — Maximum likelihood phylogeny of 19 antipatharian taxa, and maps of the complete mitochondrial genomes. [file peerj-13-18731-s003.docx]

**Hawaiian black coral (Antipatharia) complete mitochondrial genomes have limited phylogenetic signal for taxonomic resolution of species**

Supplementary Figures


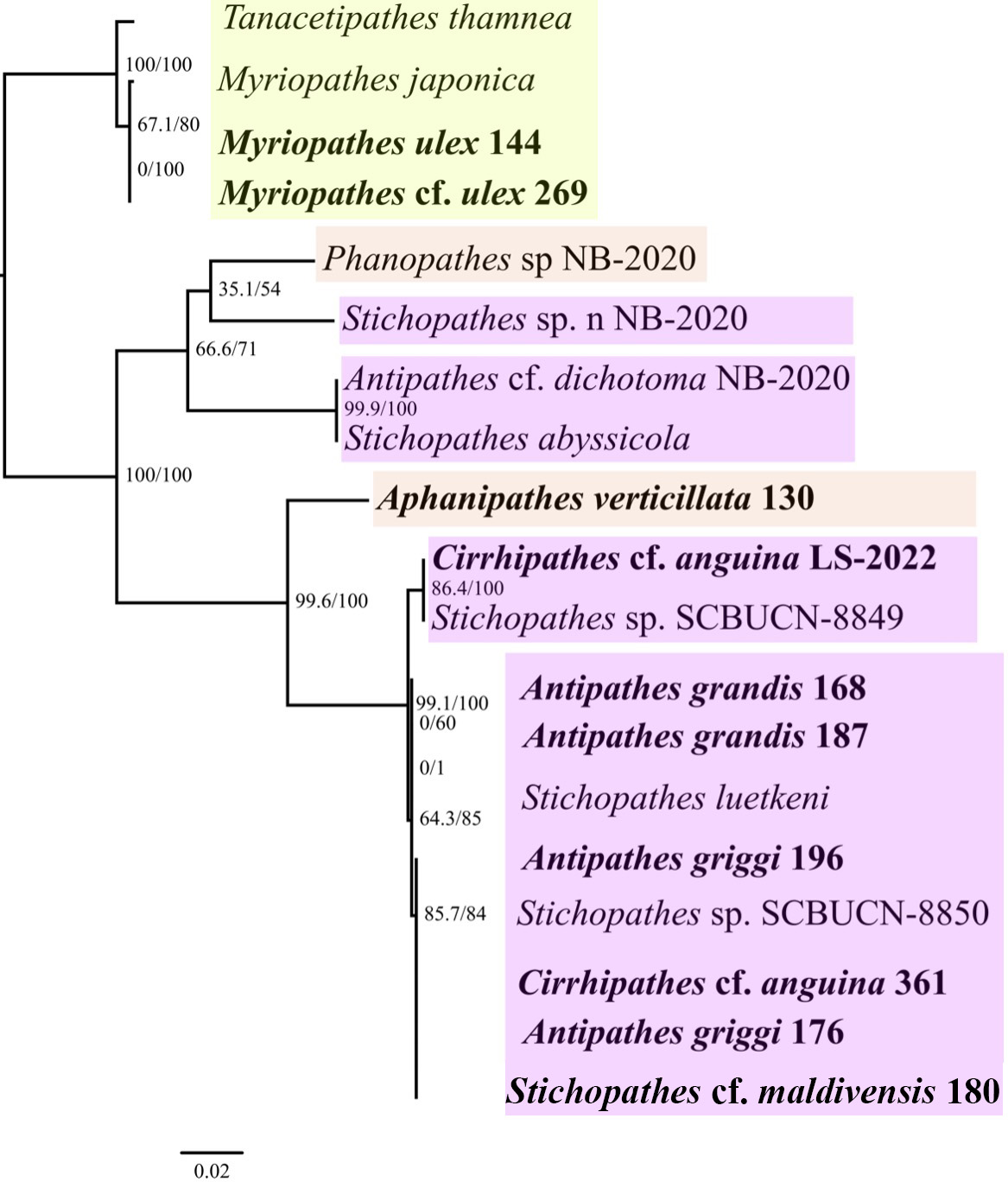


Figure S1. Maximum likelihood phylogeny of 19 antipatharian taxa belonging to three families: Myriopathidae (green), Antipathidae (pink), and Aphanipathidae (brown). The IQ-TREE inferred phylogeny is based on *ATP6* (pairwise identity: 92.6%; identical sites: 76.4%). ʻĒkaha kū mona sequenced from Hawaiʻi are bolded. Branch lengths are relative to genetic divergence, and values at each node represent SH-aLRT /ultrafast bootstrap values.


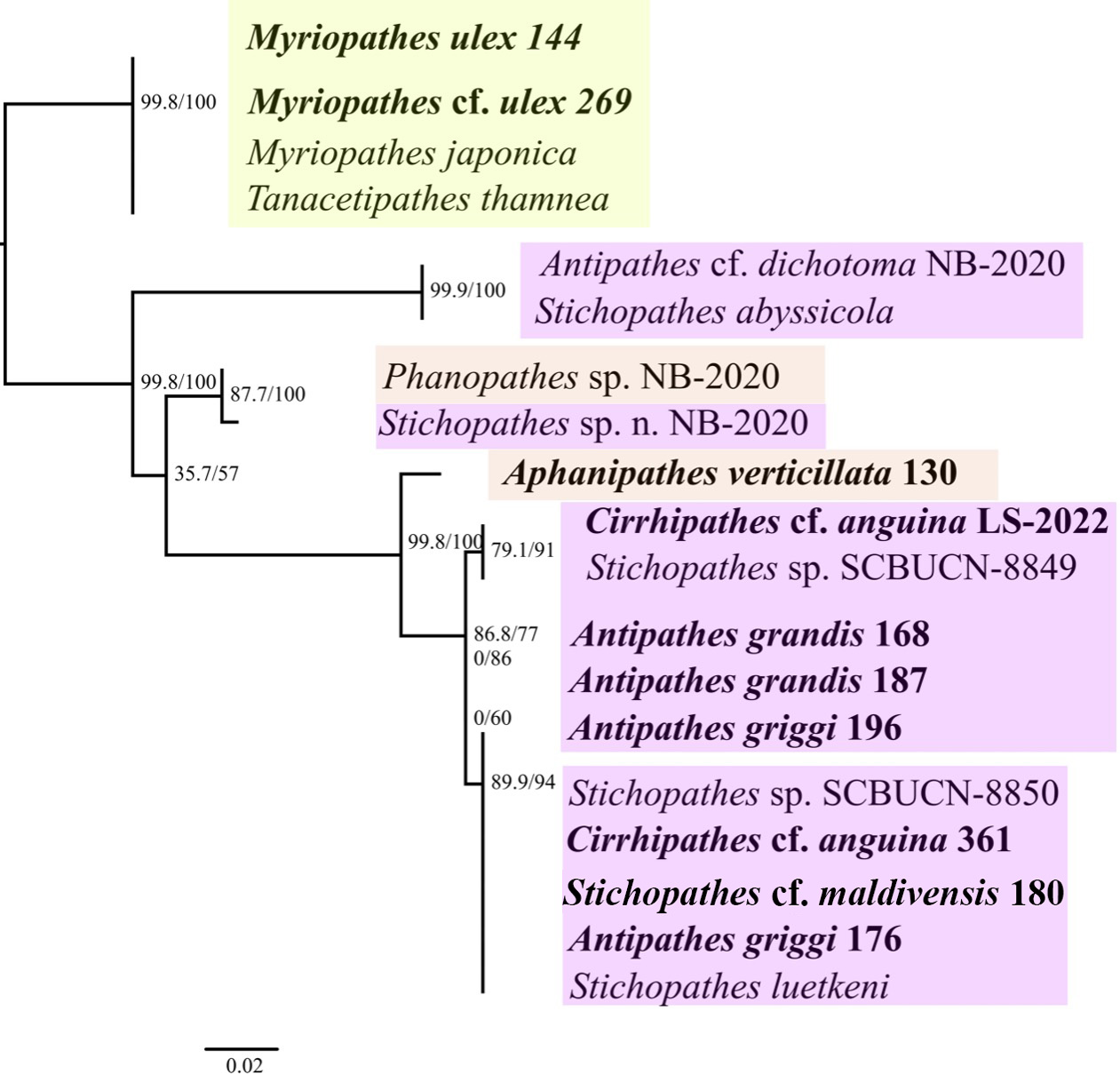


Figure S2. Maximum likelihood phylogeny of 19 antipatharian taxa belonging to three families: Myriopathidae (green), Antipathidae (pink), and Aphanipathidae (brown). The IQ-TREE inferred phylogeny is based on *ATP8* (pairwise identity: 91.8%; identical sites: 76.5%). ʻĒkaha kū mona sequenced from Hawaiʻi are bolded. Branch lengths are relative to genetic divergence, and values at each node represent SH-aLRT /ultrafast bootstrap values.


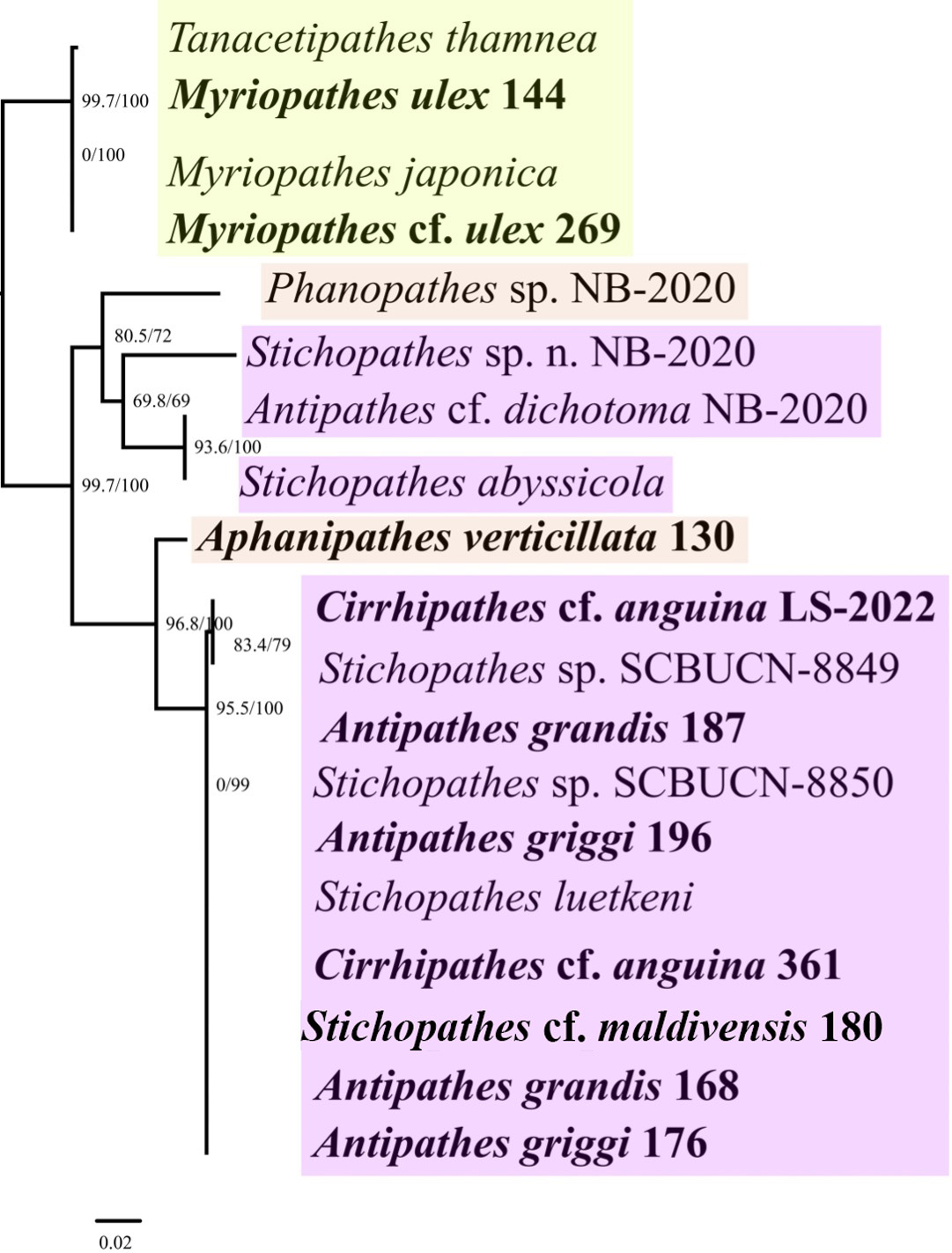


Figure S3. Maximum likelihood phylogeny of 19 antipatharian taxa belonging to three families: Myriopathidae (green), Antipathidae (pink), and Aphanipathidae (brown). This IQ-TREE inferred phylogeny is based on *ND3* (pairwise identity: 94.1%; identical sites: 81.2%). ʻĒkaha kū mona sequenced from Hawaiʻi are bolded. Branch lengths are relative to genetic divergence, and values at each node represent SH-aLRT /ultrafast bootstrap values.


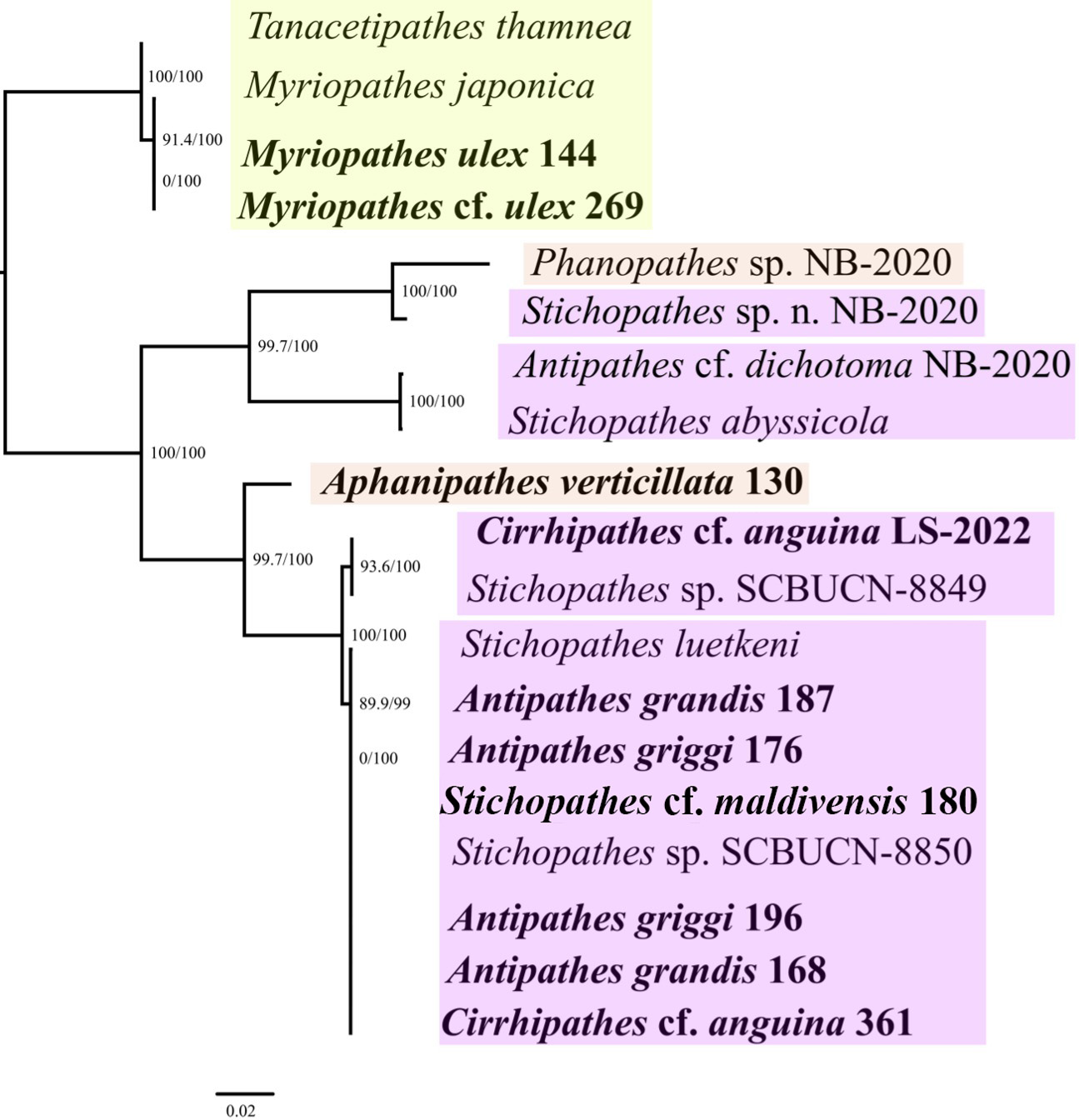


Figure S4. Maximum likelihood phylogeny of 19 antipatharian taxa belonging to three families: Myriopathidae (green), Antipathidae (pink), and Aphanipathidae (brown). That IQ-TREE inferred phylogeny is based on *ND4* (pairwise identity: 92.1%; identical sites: 76.4%). ʻĒkaha kū mona sequenced from Hawaiʻi are bolded. Branch lengths are relative to genetic divergence, and values at each node represent SH-aLRT /ultrafast bootstrap values.


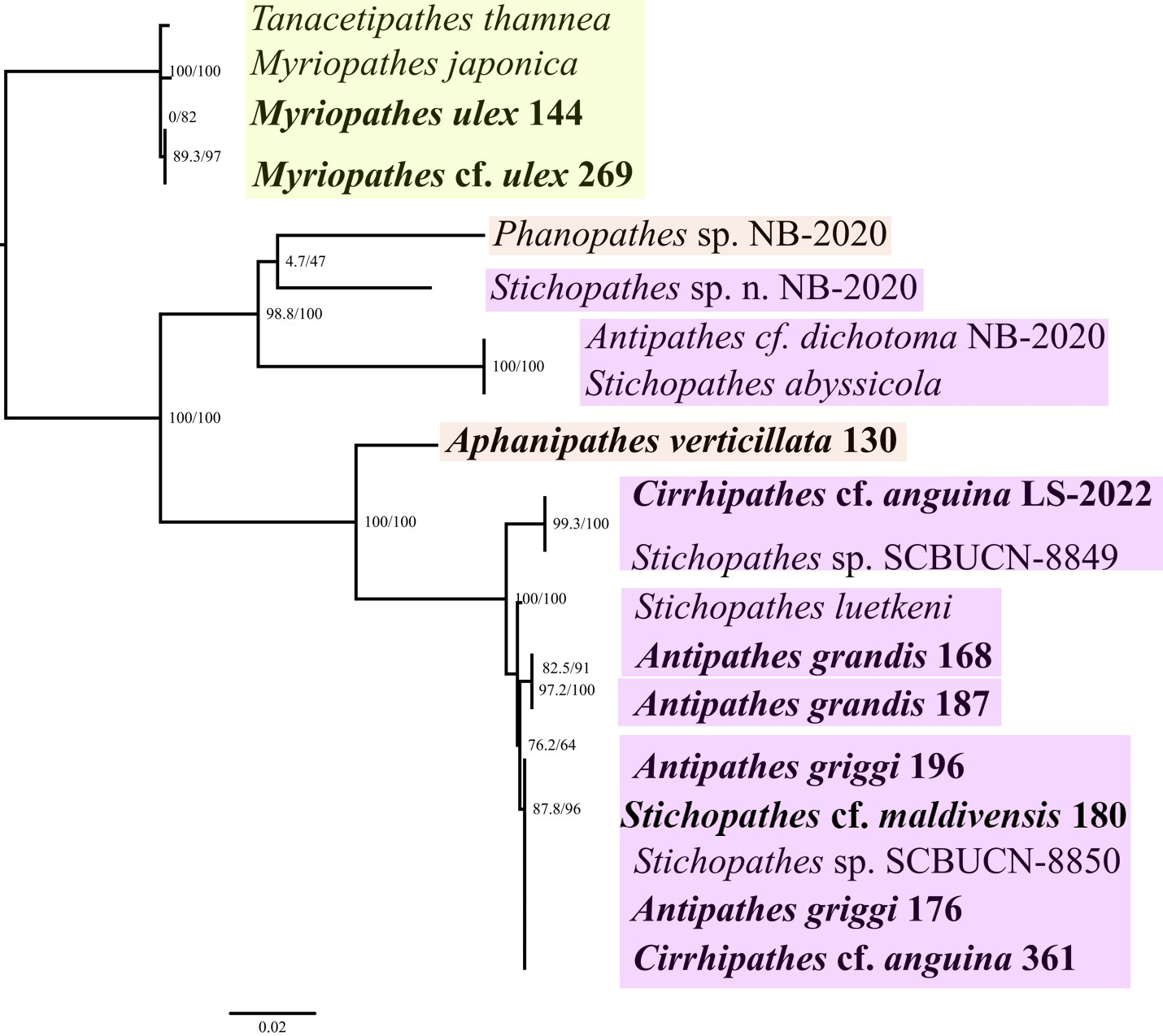


Figure S5. Maximum likelihood phylogeny of 19 antipatharian taxa belonging to three families: Myriopathidae (green), Antipathidae (pink), and Aphanipathidae (brown). The IQ-TREE inferred phylogeny is based on *ND5* (pairwise identity: 77.1%; identical sites: 36.1%). ʻĒkaha kū mona sequenced from Hawaiʻi are bolded. Branch lengths are relative to genetic divergence, and values at each node represent SH-aLRT /ultrafast bootstrap values.


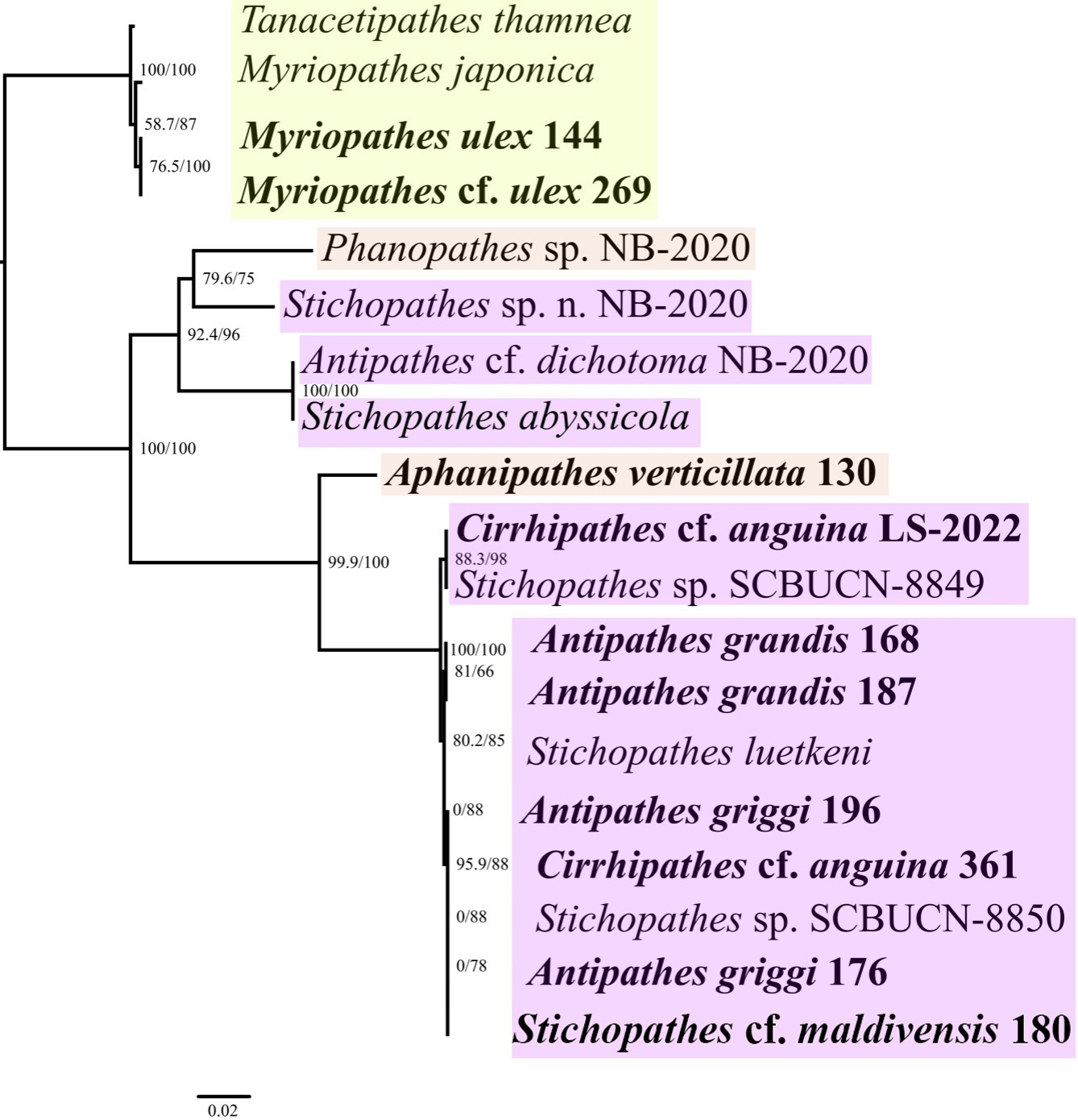


Figure S6. Maximum likelihood phylogeny of 19 antipatharian taxa belonging to three families: Myriopathidae (green), Antipathidae (pink), and Aphanipathidae (brown). This IQ-TREE inferred phylogeny is based on *COX1* (pairwise identity: 85.3%; identical sites%: 47.4%). ʻĒkaha kū mona sequenced from Hawaiʻi are bolded. Branch lengths are relative to genetic divergence, and values at each node represent SH-aLRT /ultrafast bootstrap values.


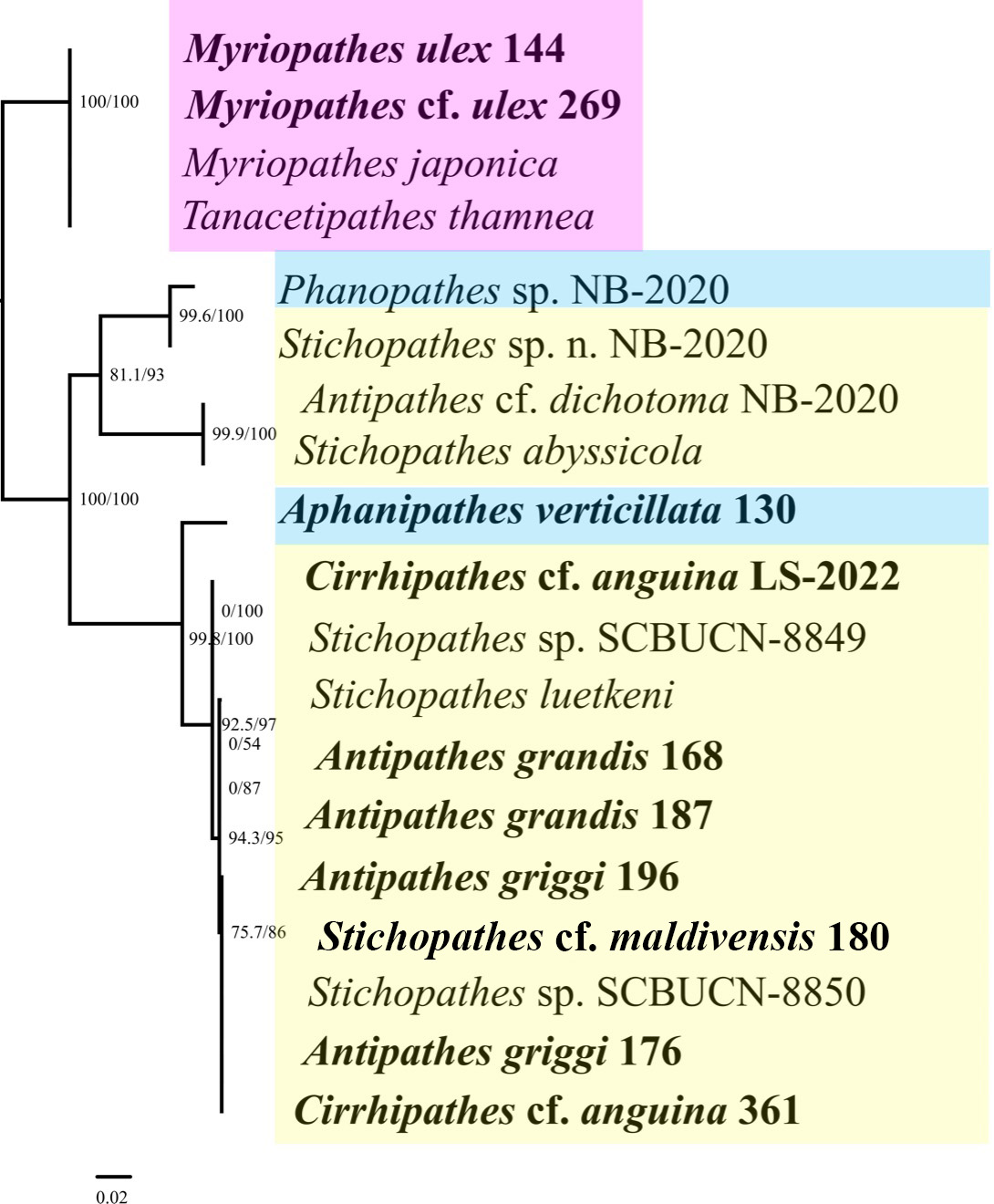


Figure S7. Maximum likelihood phylogeny of 19 antipatharian taxa belonging to three families: Myriopathidae (green), Antipathidae (pink), and Aphanipathidae (brown). This IQ-TREE inferred phylogeny is based on *COX2* (pairwise identity: 92.6%; identical sites: 78.7%). ʻĒkaha kū mona sequenced from Hawaiʻi are bolded. Branch lengths are relative to genetic divergence, and values at each node represent SH-aLRT /ultrafast bootstrap values.

**
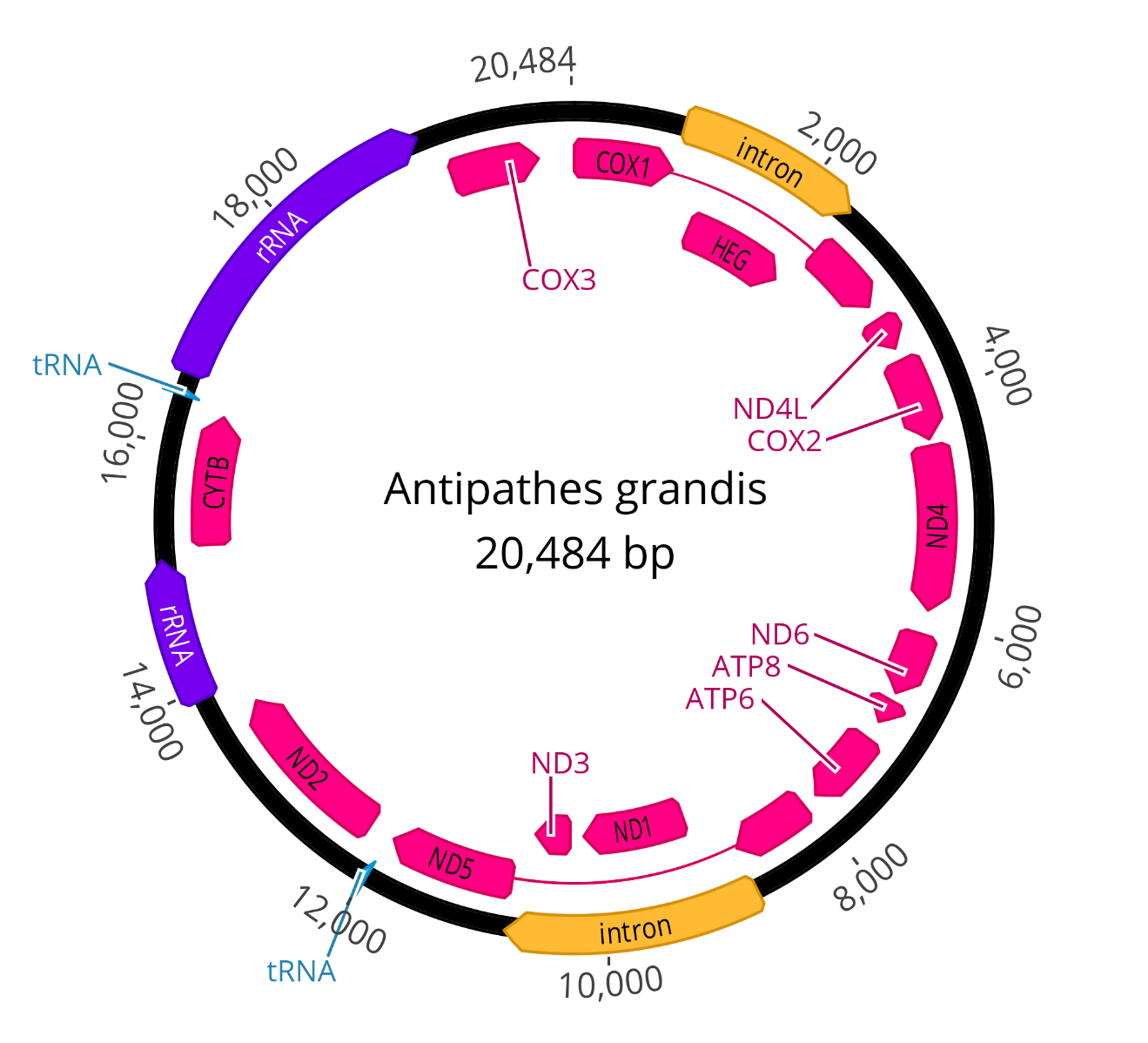
**

Figure S8. Map of the complete mitochondrial genome of *Antipathes* *grandis* (#168), drawn by Geneious Prime version 2023.2.1 (<https://www.geneious.com>).


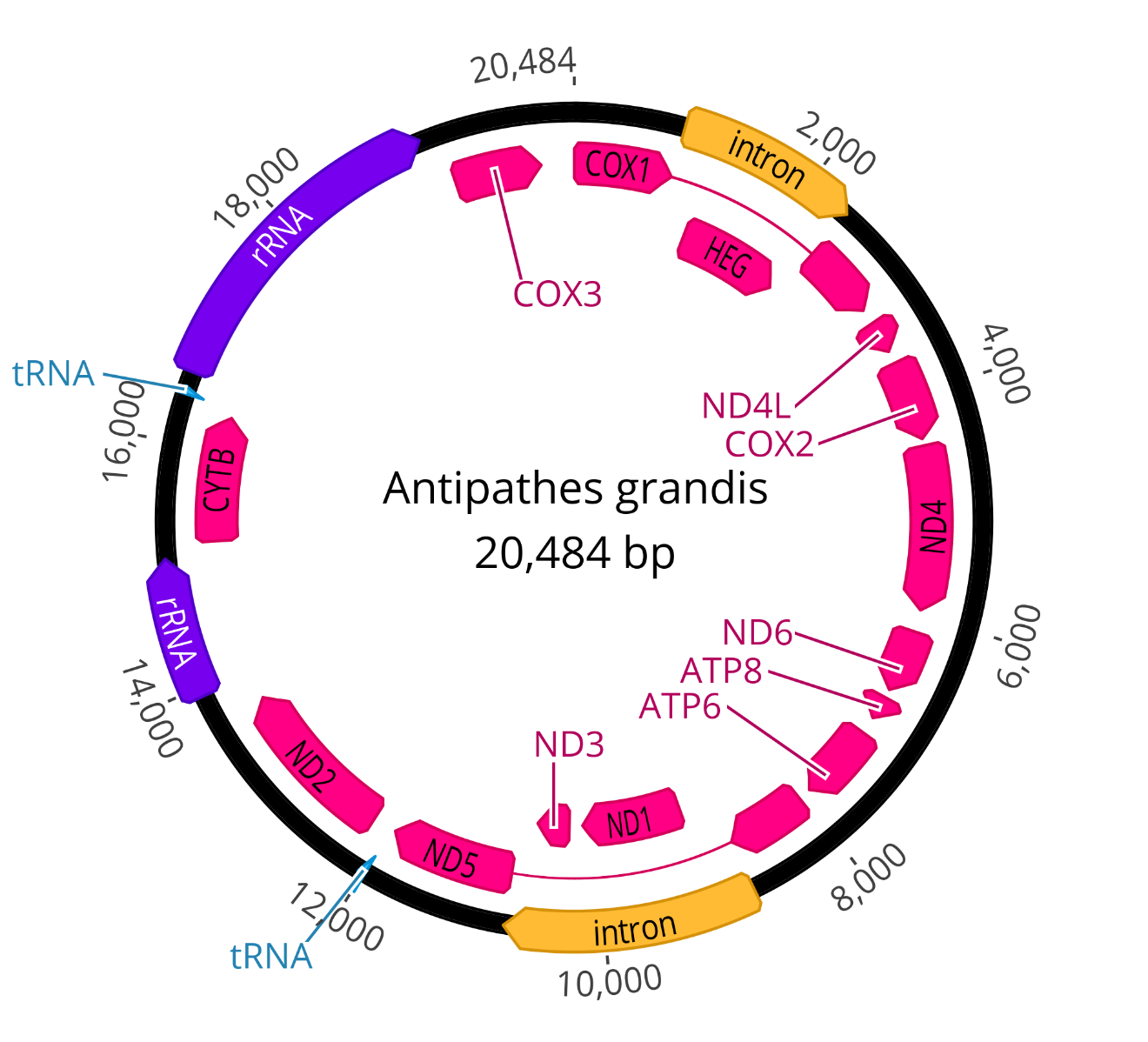


Figure S9. Map of the complete mitochondrial genome of *Antipathes grandis* (#187), drawn by Geneious Prime version 2023.2.1 (<https://www.geneious.com>).


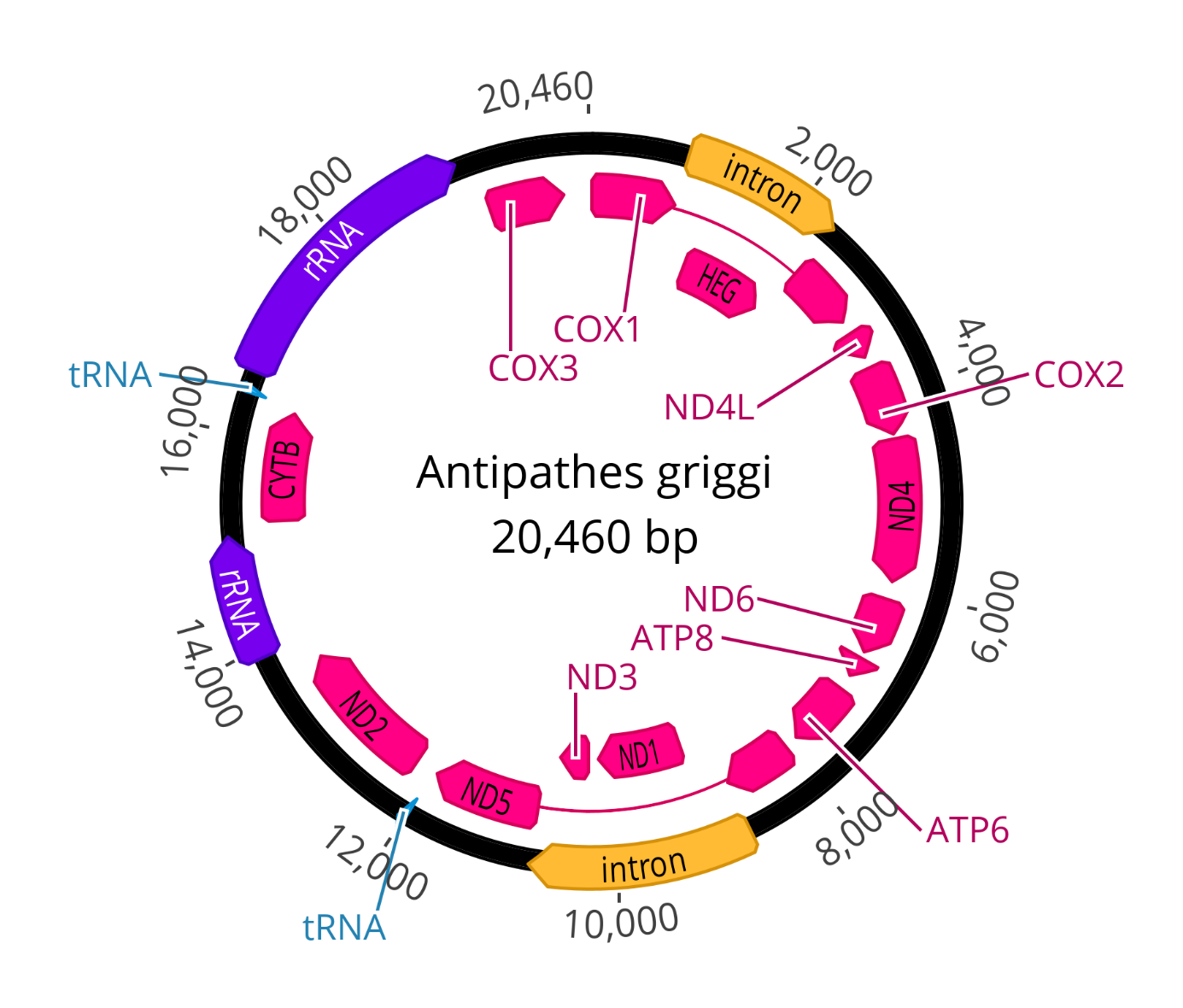


Figure S10. Map of the complete mitochondrial genome of *Antipathes griggi* (#176), drawn by Geneious Prime version 2023.2.1 (<https://www.geneious.com>).


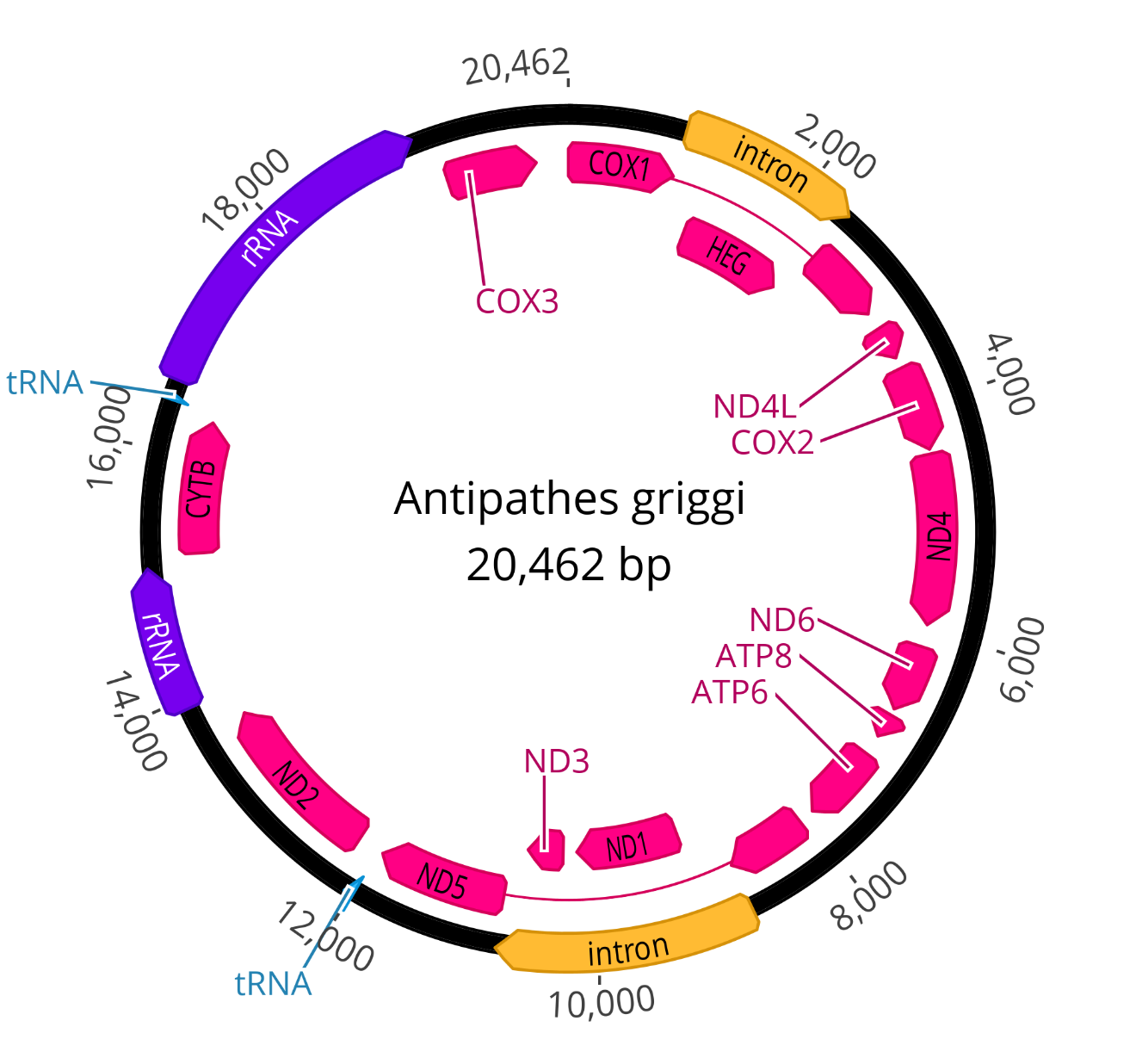


Figure S11. Map of the complete mitochondrial genome of *Antipathes griggi* (#196), drawn by Geneious Prime version 2023.2.1 (<https://www.geneious.com>).


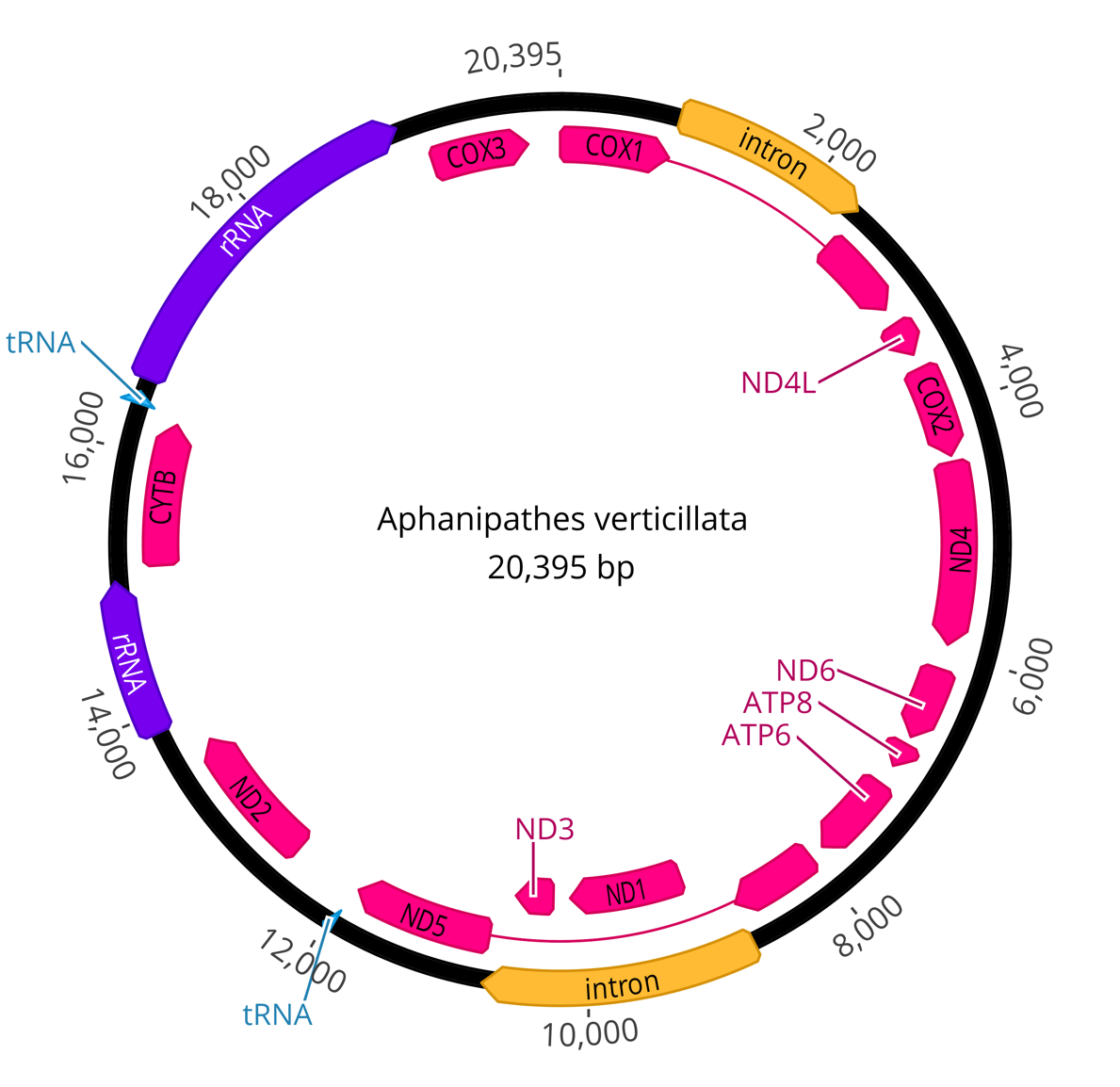


Figure S12. Map of the complete mitochondrial genome of *Aphanipathes* *verticillata* (#130), drawn by Geneious Prime version 2023.2.1 (<https://www.geneious.com>).


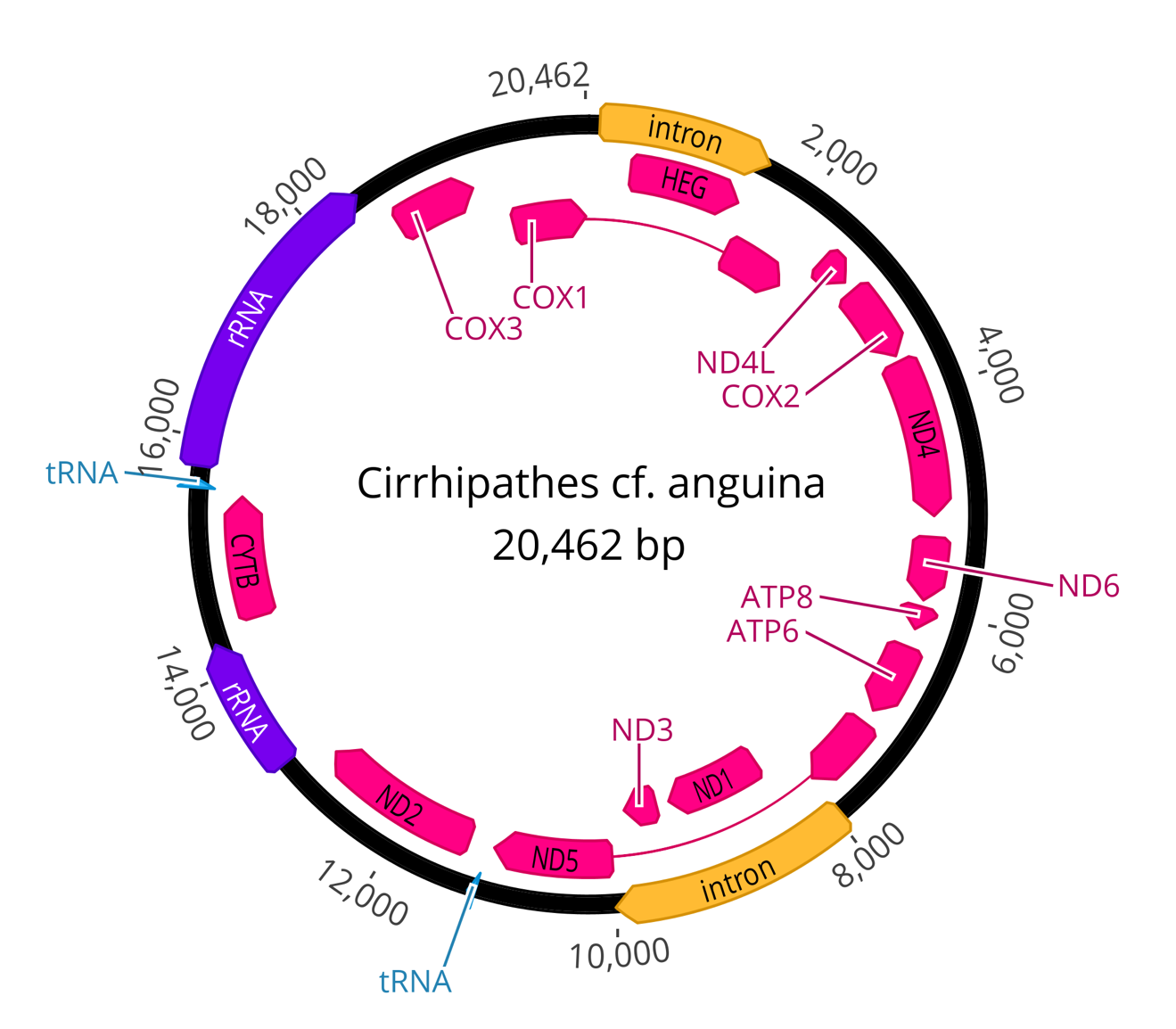


Figure S13. Map of the complete mitochondrial genome of *Cirrhipathes cf. anguina* (#361), drawn by Geneious Prime version 2023.2.1 (<https://www.geneious.com>).


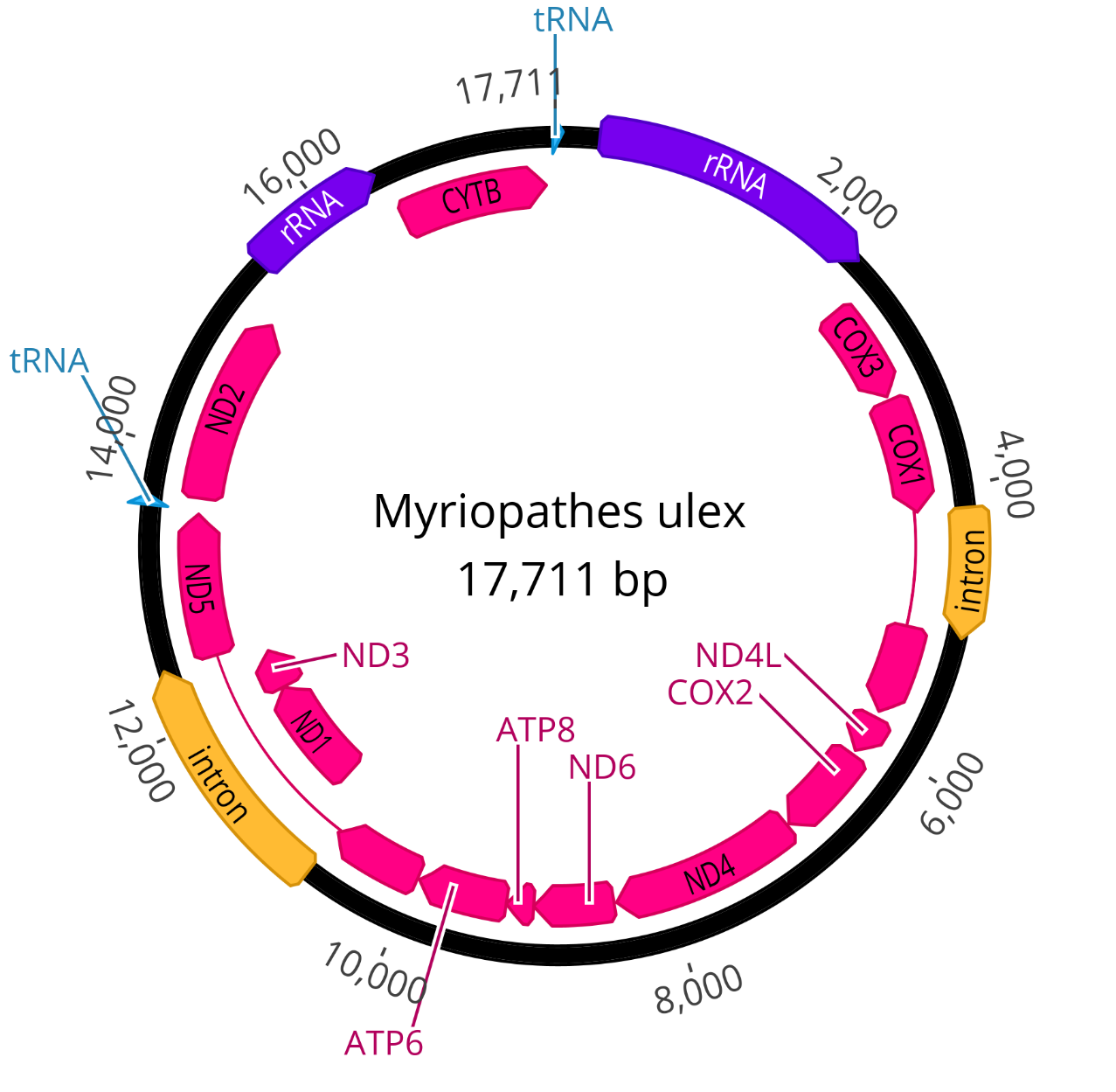


Figure S14. Map of the complete mitochondrial genome of *Myriopathes ulex* (#144), drawn by Geneious Prime version 2023.2.1 (<https://www.geneious.com>).


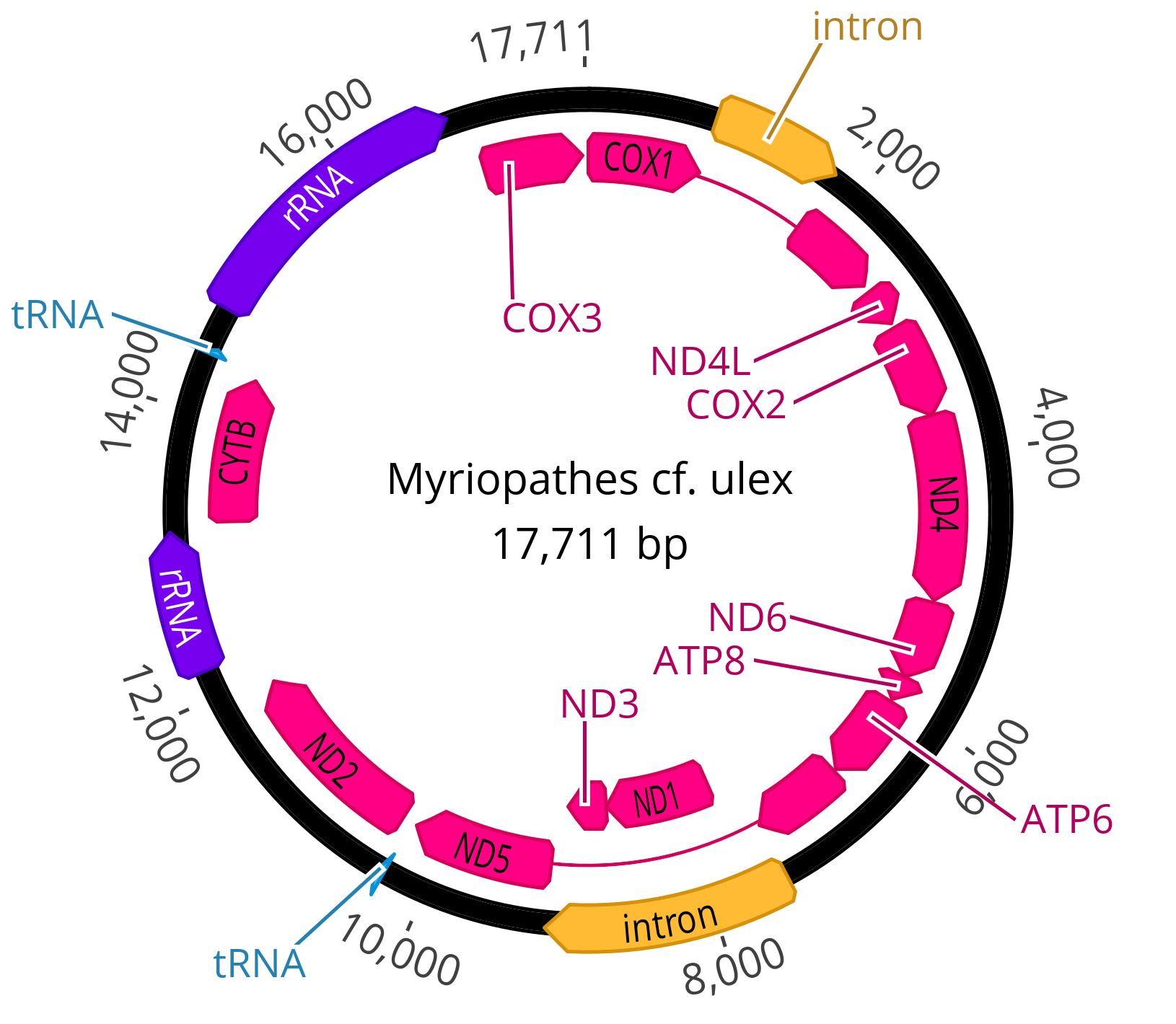


Figure S15. Map of the complete mitochondrial genome of *Myriopathes* cf. *ulex* (#269), drawn by Geneious Prime version 2023.2.1 (<https://www.geneious.com>).


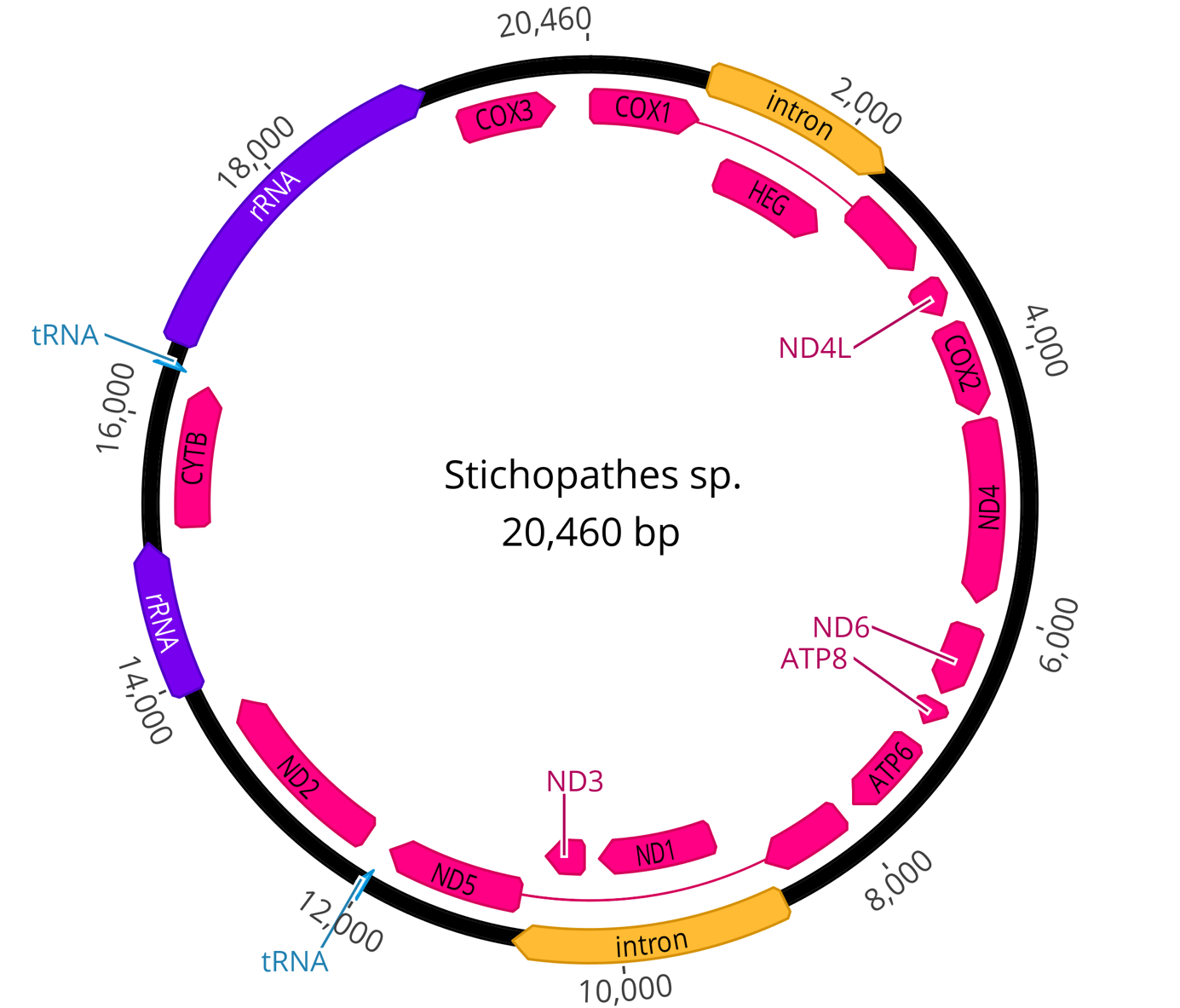


Figure S16. Map of the complete mitochondrial genome of *Stichopathes* cf. *maldivensis* (#180), drawn by Geneious Prime version 2023.2.1 (<https://www.geneious.com>).
